# Bullying and sexual harassment of medical and nursing students, in relation to stress, burnout and intention to dropout

**DOI:** 10.5116/ijme.6a31.6bd0

**Published:** 2026-07-02

**Authors:** Caroline Olsson, Anna Toropova, Irene Jensen, Christina Björklund

**Affiliations:** 1 Unit of Intervention and Implementation Research for Worker Health, Institute of Environmental Medicine, Karolinska Institutet, Stockholm, Sweden

**Keywords:** Bullying, sexual harassment, medical students, nursing students, stress, burnout, dropout intention, student mistreatment, medical education, nursing education

## Abstract

**Objectives:**

This study examines the prevalence and impact of bullying and sexual harassment among students in nursing and medical education programmes in Sweden.

**Methods:**

This cross-sectional survey targeted students from 38 universities. A total of 18,582 individuals responded to the questionnaire, yielding a 25% response rate. The sample included students enrolled in nursing (N = 1,083) and medical (N = 431) programmes. Data were analysed using descriptive statistics and two-sample t-tests.

**Results:**

Among female students who experienced bullying, higher levels of stress ( t _(1188)_ = 4.91, p < .001), burnout (t_(1188)_ = 5.83, p < .001), and intention to quit studies ( t _(1186)_ = 4.30, p < .001) were reported. Bullied male students showed elevated stress ( t _(317)_ = 3.15, p = .002), burnout ( t _(317)_ = 3.49, p < .001), and intention to quit ( t _(316)_ = 3.67, p < .001). Female students who experienced sexual harassment reported increased stress ( t _(1185) _= 4.02, p < .001), burnout ( t _(1185) _= 4.10, p < .001), and intention to quit ( t _(1184)_ = 2.73, p = .006). In contrast, sexually harassed male students reported higher stress ( t _(314)_ = 2.04, p = .042), but no significant differences in burnout ( t _(314)_ = 0.80, p = .425) or intention to quit ( t _(314)_ = 1.86, p = .064). Students from the nursing- and medical programme reported a higher prevalence of bullying and sexual harassment than other students.

**Conclusions:**

Given the high prevalence and detrimental effects of bullying and sexual harassment in nursing and medical education, targeted interventions are needed to prevent and address these behaviours.

## Introduction

Numerous studies have showed that the nursing ^1^ and medical educational^2^^-^^4^ environments are not immune to the challenges of bullying and sexual harassment. These behaviours impact both individual students and the educational context, and in the long run it might affect the availability of healthcare professionals, as it increase the intention to dropout of nursing education^5^^, ^^6^ or medical school.^7^ The definition of bullying is related to situations where an individual repeatedly and over a prolonged period of time is exposed to harassing behaviour from one or more individuals and has difficulties in defending themselves^8^^, ^^9^ Sexual harassment, on the other hand, can be described as unwelcome sexual initiatives, and other verbal and physical harassment of a sexual nature.^10^ A considerable volume of research exists on the prevalence of bullying and sexual harassment within nursing and medical environments. However, several reviews on the topic have demonstrated variation in the prevalence rate reported, and those can often be attributed to the type of measurement scale used, different definitions of bullying and sexual harassment, and contextual differences. As an example, an integrative literature review of the bullying of nursing students stated that the bullying prevalence varied from 9–96%^6^, and a systematic review of female nursing students during clinical placement placed the prevalence rates at between 7.2-68 %. ^11^ For medical students, a review and meta-analysis revealed that 33.3 % of medical students reported sexual harassment.^4^

Gender imbalance is related to risk of harassment.^12^^,^^13^ The gender composition differs between nursing education and medical education, where nursing has traditionally been a female dominated profession and continues to be so,^14^ whereas women have been underrepresented in medical education until recently.^15^ In Sweden in 2023, there were 5,124 women registered as students in a medical programme and 3,438 male students, while at nursing school there were 13,145 women and 2,209 men.^16^ Previous research of registered nurses and nursing students found that sexual harassment was more frequently reported by women compared to men, but that men had higher prevalence in terms of severe cases.^17^ Several studies have shown that women in medical education are more exposed to different forms of inappropriate behaviour than men, and that men are more often reported to be perpetrators.^3^^,^^18^^-^^20^ Bullying and sexual harassment are significant problems, and several studies have aimed to identify the mechanisms behind their persistent nature. It has been suggested that the medical field is characterised by male dominated hierarchical organisations,^21^^-^^23^ closed networks and a silence culture that punish those how report inappropriate behaviour.^2^ Even if the number of women in medicine has increased and is gender balanced in many countries now, old gender norms still persist.^24^ The nursing profession, on the other hand, is still mostly composed of women, and this has been understood in terms of caring and femininity and a place where men can be met with superstition regarding their professional choice.^25^^,^^26^ Both nursing and medical education take place at the university and in the health care system, which means that students are part of two contexts that often are linked. In Sweden there are seven universities that provide the medical programme, and all these also provide nursing education. Students from the two programmes share their campus experiences and some parts of the education are interprofessional with several integrated stages to gain interprofessional competence. As an example, students from the two programmes take part in interprofessional activities during their clinical practise.^27^ A study from Sweden among medical students identified lectures and coffee breaks as common places for sexual- and gender-related harassment, along with clinical placements.^20^ During clinical placements, both nursing and medical students report the perpetrators as patients, their relatives or clinical staff.^4^^, ^^28^^-^^30^

### Consequences

It has been recognised that bullying and sexual harassment have negative consequences for students on an individual level, as well as for the universities with increased risk of students dropping out of their education.^31^ A Swedish study investigating sexual harassment and sexual violence found associations between increased risk of depression and anxiety for female students,^32^ and two reviews covering consequences of bullying and sexual harassment of nursing students show an increased risk of mental health problems, including burnout symptoms,^6^^, ^^33^ For medical students, sexual harassment^18^ or other mistreatment^34^ has been found to be associated with increased rates of feelings of burnout. For instance, medical students who said that they had been sexual harassed by patients reported higher levels of feelings of burnout (21% for women and 5% for men) compared to students who did not report sexual harassment.^18^According to a systematic review focused on higher education among female students,^31^ university dropout, was identified as a consequence of sexual assault.

Until now, there has been a lack of national data covering Swedish medical and nursing students’ vulnerability to bullying, sexual harassment, stress and burnout, meaning that there is a knowledge gap regarding students within the Swedish context. The aim of this study was therefore to examine the prevalence of bullying and sexual harassment of nursing and medical students and to investigate the relationship between these risks and perceived stress, burnout symptoms and intention to drop out of education.

## Methods

### Study design

This study is part of a collaboration programme between four universities in Sweden (Karolinska Institutet, Royal Institute of Technology, Malmö University and University of Gothenburg). The questionnaire was developed by researchers from these universities in collaboration with Statistics Sweden. The questionnaire mainly consists of validated scales. Survey data on students, PhD students, and employees from 38 universities that are part of the Association of Swedish Higher Education Institutions (SUHF) were collected by Statistics Sweden between March-July 2021. Participants were randomly selected and extracted from the Swedish higher education register.^35^ The survey was pilot tested with a subgroup of the target population, confirming that the questions were clear and suitable. Cronbach’s alpha showed high reliability in both the pilot and the main study. As no changes were needed, the final questionnaire was used in its original form. The study was approved by the Swedish Ethical Review Authority and with adherence to the Declaration of Helsinki’s ethical guidelines. The web-based questionnaire was distributed by Statistic Sweden including information that it was voluntary to participate, and that it was possible to withdraw from participation at any time. Informed consent was given by participants by sending in the questionnaire. Statistic Sweden processed and depersonalised the data before delivering it to Karolinska Institutet for analysis which guaranteed anonymity for the participants**. **A total of 372 396 students were eligible for inclusion, of which 75 927 students were randomly selected to participate. An invitation letter was sent by post to the selected participants, which provided information about the study and login details to the online survey. Three reminder letters were sent to non-responders during the data collection period.A total of 18 582 participants filled in the questionnaire and the response rate of 25.2 percent.

### Participants

In the current study, a subgroup of the data from the main study was used, consisting of individuals who at time of data collection were “students in the nursing or the medical programme”. Descriptives are presented in Table 1. Data consist of 431 medical students (men 162/women 269) from all seven medical faculties in Sweden that provide medical education as well as 1083 nursing students (men 157/women 926) from 25 universities or university colleges.

### Variables and measures

All included variables are validated scales from the Copenhagen Psychosocial Questionnaire (COPSOQ).^36^ The scales have been extensively validated in different work-settings and occupational groups in Sweden and elsewhere.

### Outcome variables

The first outcome variable measured burnout symptoms. Participants were asked: "During the last 4 weeks, how often have you": (1) been emotionally exhausted, (2) been physically exhausted, or (3) felt worn out.  Response alternatives were: (1) All the time, (2) A large part of the time, (3) Part of the time, (4) A small part of the time, and (5) Not at all.  An index denoting burnout symptoms was created by taking the mean value of the above three items. Reliability statistics of the burnout index, Cronbach’s alpha, was .86, indicating good reliability.

The second outcome variable measured stress. Participants were asked: “During the last 4 weeks, how often have you": (1) found it difficult to relax, (2) been irritable, (3) been tense. Response alternatives were: (1) All the time, (2) A large part of the time, (3) Part of the time, (4) A small part of the time, and (5) Not at all.  An index describing stress symptoms was created by taking the mean value of these three items. Reliability statistics of the stress index, Cronbach’s alpha, was .82, indicating good reliability.

The third outcome variable assessed intention to quit the studies. Participants were asked: “Have you considered resigning from your current studies?”. Response alternatives were: (1) Never, (2) A few times, (3) Sometimes, (4) Often, and (5) Many times. 

### Explanatory variables

Workplace bullying was measured with one question, “Have you been exposed to bullying at your place of study during the last 12 months?”, with the accompanying explanation: “Bullying is when a person is repeatedly exposed to unpleasant or degrading treatment and finds it difficult to defend themselves”. Response alternatives were: (1) Yes, daily, (2) Yes, every week, (3) Yes, every month, (4) Yes, a few times, and (5) No.  Sexual harassment was measured with one question: “During the last 12 months, have you been exposed to undesired sexual attention at your workplace”. Response alternatives were: (1) Yes, daily, (2) Yes, every week, (3) Yes, every month, (4) Yes, a few times, and (5) No. Questions regarding the relationship to the aggressor were measured with two questions regarding the aggressor’s gender and position with the response alternatives teacher/supervisor, student, patient or other.

### Analysis

Bullying and sexual harassment variables were dichotomised. The response alternatives 1 to 4 were recoded into ‘Yes’ and 5 into ‘No’ as recommended in COPSOQ^36^. The response alternatives for stress and burnout were transformed to 0 to 100, as recommended in COPSOQ^36^: (1) ‘All the time’ was transformed to 100, (2) ‘A large part of the time’ was transformed to 75, (3) ‘Part of the time’ was transformed to 50 (4), ‘A small part of the time’ was transformed to 25, and (5) ‘Not at all’ was transformed to 0.

A series of statistical analyses were performed using version 27.0 of the IBM SPSS program. First, descriptive statistical analyses were performed, including means and standard deviations for continuous variables, percentages for categorical variables. Cross-tabulations were performed to demonstrate how the variables differ across various subgroups, such as for e.g. gender. Two-sample t-tests were performed to compare stress levels, burnout symptoms and intention to quit in the bullied vs. non-bullied groups of students as well as the harassed vs. non-harassed groups of students. Results were considered statistically significant at p<.05.

## Results

### Descriptive statistics

Participant demographic characteristics in medical and nursing programmes as well as reported bullying/sexual harassment are presented in Table 1.

**Table 1:** Participant demographic characteristics and reported bullying and sexual harassment

Variable	Medical programme(N=431)		Nursing programme (N=1083)	
	Men	Women	Men	Women
Gender,N (%)	162 (37.6)	269 (62.4)	157 (14.5)	926 (85.5)
Age,M (SD)	26.71 (5.59)	25.42 (4.48)	32.55 (9.40)	29.37 (8.25)
Swedish background,N (%)	142 (87.7)	225 (83.6)	124 (79.0)	780 (84.2)
Subjectedto bullying, N (%)	12 (7.4)	28 (10.4)	14 (8.9)	77 (8.3)
Subjectedto sexual harassment, N (%)	10 (6.2)	36 (13.4)	16 (10.3)	68 (7.4)

As can be observed, around 10% of women in the medical programme were subjected to bullying compared to around 7% of men. With regards to sexual harassment, the proportion of women subjected was around 13% while the proportion of men sexually harassed was 6%. The highest prevalence of both bullying and sexual harassment was reported by female students in medical education (10.4/13.4%). For male students, the prevalence was highest in nursing education with 8.9 and 10.3% for bullying sexual harassment, respectively. We also conducted additional analyses to compare these results with the total cohort of students from the national data collection of which this study was a part. Among students in all higher education programmes, except for those in nursing- and medical programmes, women reported 9.1% for bullying and 4.5% for sexual harassment and the equivalent numbers for men were 6.0% for bullying and 2.3% for sexual harassment demonstrating that the prevalence is higher in medical- and nursing programmes compared to other higher education programmes.

### Bullying within medical and nursing programmes

#### Gender of the bullied and the aggressor

The majority of bullied men reported that the aggressors were women, followed by aggressors being from both sexes. The majority of bullied women also reported that the aggressors were women, followed by aggressors being men.

#### The relationship between the bullying victim and the aggressor

The victim of bullying was asked to report what relationship they had to the aggressor (Table 2).

Results show that the most common aggressor both for men and women victims of bullying was another student (s) at the study place. The second most common aggressor for each gender was a teacher /supervisor. Women reported to a higher extent than men that the teacher/supervisors were the aggressor, whereas men reported the alternative Other as the aggressor more frequently.

**Table 2:** The relationship between the bullying victim and the aggressor

Gender of student bullied		Teacher/supervisor	Student	Patient	Other	Total ^b^
Men	N (%)^ a^	10 (38)	14 (54)	4 (15)	4 (15)	36
Women	N (%)^ a^	54 (51)	56 (53)	24 (23)	7 (7)	141
Total	N (%)^ a^	64 (49)	70 (53)	28 (21)	11 (8)	173

^a ^% within the gender. A total number of men= 26, A total number of women= 105.

^b ^This number can exceed 100% since victims can report more than one aggressor

### Sexual harassment within medical and nursing programmes

#### Gender of the sexually harassed and the aggressor

The largest proportion of men reported that the aggressors subjecting them to sexual harassment were women (75%) and an equal proportion of men (around 8%) named men, women and men, or others as aggressors. The largest proportion of women reported that the aggressors exposing them to sexual harassment were men (around 86%). Around 5% of women stated that both women and men were the aggressors.

#### The relationship between the victim of sexual harassment and the aggressor

The victim of sexual harassment was asked to report what relationship they had to the aggressor (Table 3). The respondent could report more than one aggressor.

Results show that the most common aggressor subjecting men to sexual harassment was a student while women named patients as the most common aggressor. The next most common aggressor for men was patients while it was students for women.

**Table 3:** The relationship between the sexual harassment victim and the aggressor

Gender of student sexually harassed		Teacher/supervisor	Student	Patient	Other	Total ^b^
Men	N (%) ^a^	4 (15)	12 (46)	10 (38)	4 (19)	30
Women	N (%) ^a^	28 (27)	44 (42)	60 (58)	18 (17)	150
Total	N (%) ^a^	32 (25)	56 (43)	70 (54)	22 (17)	173

^a ^% within the gender. A total number of men= 26, A total number of women= 104.

^b ^This number can exceed 100% since victims can report more than one aggressor

### Bullying association with stress, burnout and intention to quit

Overall, the bullied students reported higher levels of stress and burnout symptoms, as well as expressed a stronger intention to quit studies.

Among the female students, t-test results indicated that those subjected to bullying reported statistically significantly higher levels of stress symptoms *t*_(1188)_=4.91, p<.001, mean difference 11.47, 95% CI[6.88, 16.05], burnout symptoms *t*_(1188)_=5.83, p<.001, mean difference 15.07, 95% CI[10.00, 20.14], as well as expressed a stronger intention to quit studies *t*_(1186)_=4.30, p<.001, mean difference 0.46, 95% CI [0.25, 0.68].  Among the male students, t-test results indicated that those subjected to bullying reported statistically significantly higher levels of stress compared to non-bullied ones *t*_(317)_=3.15, p=.002, mean difference 14.25, 95% CI[5.36, 23.14]. Male bullied students also reported statistically significant burnout symptoms *t*_(317)_=3.49, p <.001, mean difference 17.26, 95 CI[7.52, 26.99]. Finally, male bullied students expressed a stronger intention to quit studies: *t*_(316)_=3.67, p<.001, mean difference 0.76, 95% CI[0.35, 1.17].

#### Sexual harassment association with stress, burnout and intention to quit

Overall, sexually harassed students experienced higher levels of stress and burnout symptoms, as well as expressed a stronger intention to quit studies.

Among female students, according to independent samples t-test results, those exposed to sexual harassment reported statistically significantly higher levels of stress: *t*_(1185)_=4.02, p<.001, mean difference 9.48, 95% CI[4.85, 14.10] and burnout: *t*_(1185)_=4.10, p<.001, mean difference 10.71, 95% CI[5.58, 15.84].  Female bullied students also expressed a stronger intention to quit studies *t*_(1184)_=2.73, p=.006, mean difference 0.30, 95% CI [0.08, 0.51].

Among the male students, there was a statistically significant difference in stress levels between the sexually harassed and non-harassed groups *t*_(314)_=2.04, p=.042, with those exposed to sexual harassment reporting statistically significantly higher levels of stress mean difference 9.35, 95% CI[0.35, 18.34]. With regards to burnout symptoms, there was no statistically significant difference between sexually harassed and non-harassed groups *t*_(314)_=0.80, p=.425.  Finally, men subjected to sexual harassment did not express a stronger intention to quit studies *t*_(314)_=1.86, p=.064.

### Differences in stress, burnout and intention to quit by program

Women in the medial programme reported higher levels of stress and burnout than men, as well as somewhat stronger intention to drop out of their education. Bullying in the nursing programme was reported by nearly equal proportions of women (about 8%) and men (nearly 9%), while the share of women subjected to sexual harassment was around 7% compared to about 10% of men. Overall, women in the nursing programme reported higher levels of stress and burnout, together with a weaker intention to quit studies, compared to men.

## Discussion

Our findings align with previous research indicating that students in medical education are more frequently exposed to bullying and sexual harassment compared to those in other academic disciplines. In all higher education programmes - excluding nursing and medicine - 9.1% of female students reported bullying and 4.5% reported sexual harassment. Among male students, the corresponding figures were 6.0% and 2.3%, respectively.

In contrast, students in the medical programme reported notably higher rates. Among female students, 10.4% experienced bullying and 13.4% reported sexual harassment, while male students reported 7.4% and 6.2%, respectively. A similar pattern was observed in the nursing programme, where 8.3% of female students reported bullying and 7.4% sexual harassment, compared to 8.9% and 10.3% among male students.

One possible explanation is that students in these programmes may be exposed to such behaviours from patients, a factor supported by our data.

Our data on sexual harassment demonstrate that male students were more exposed by women, whereas female students reported that they were most often harassed by a man. One possible explanation for these results may be that the traditional gender composition of nursing and medical educational programmes persists. A numerus of previous studies conducted in academic settings have showed similar patterns.^35^^,^^37^^,^^38^ Gender balance in working groups may even have a buffering effect on sexual harassment, Hu and colleagues suggest that medical specialist areas with a mixture of male and female doctors reduced the risk of bullying and harassment (including sexual harassment) for both men and women.^12^ In our study both male and female students were more often bullied by a woman. However, it should be noticed that gender patterns for bullying vary considerably between different studies.^39^

We were interested in consequences that can arise from bullying and sexual harassment and we found increased levels of self-reported stress for both male and female students for those who reported bullying. Expose to sexual harassment was also associated with higher rates of burn out and intention to drop out of education for female students but not for male students.

Our results indicate that, in line with previous studies, bullying and  sexual harassment are associated with  increased levels of self-reported stress and burnout symptoms, as well as the risk of dropout,^40^ especially among women.^6^^, ^^34^^, ^^41^ These findings imply negative consequences for the individual student as well as for the education and the profession.

### Strength and limitations

Data collection was conducted during the Covid-19 pandemic, which may have impacted the outcome of sexual harassment, bullying as well as mental health data. Caution should therefore be taken when comparing the results with other studies. Even if education during the pandemic became, to a large extent, digital, students from the nursing and medical programmes were not greatly affected since they still had clinical rotations in health care settings. In addition, the Swedish model, with no regular lockdown, meant that during the Covid-19 pandemic Swedish medical- and nursing education was subject to fewer changes in daily life compared with students from other settings.^42^

In this study, we used self-reported data, with single direct questions on bullying and sexual harassment. To increase the validity and comparability, we only used scales validated in a Swedish context.^36^ Still, single direct questions are known to give lower prevalence numbers than questionnaires with multiple questions to measure a phenomenon.^43^ In addition a cross-sectional study does not allow conclusions about causality or the direction of effects, we therefore strongly recommend longitudinal studies in the future to be able to establish causality between sexual harassment, burnout, and dropout intentions, overcoming the limitations of cross-sectional design.

### Implications

To reduce the risk of bullying and sexual harassment, several steps are needed. First, these behaviours should be recognised and addressed even if they are not severe because when bullying and sexual harassment can pass under the radar there is a risk that these inappropriate behaviours are seen as an inherent part of the educational milieu and become normalised.^44^ To create a positive learning environment, students need to feel safe on campus and in clinical settings.

Second, universities and clinical settings need to collaborate regarding the prevention of bullying and sexual harassment, this mean that both university staff and clinical staff need to have knowledge regarding bullying and sexual harassment. To train the staff is one way to make them prepared to take action when needed. A large proportion of the education takes place in clinical settings, and sometimes the perpetrators are patient or relatives. This means that clinical supervisors need to have knowledge of how to prevent harassment during clinical practice. When patients or relatives pass the line of appropriate behaviour, a supervisor should be able to support the student and react to inappropriate behaviour. Since they are role models, their response to different forms of mistreatment will be part of the student’s professional socialisation. In a qualitative study where medical students were asked to bring solutions to the problem of sexual harassment in medical education, they emphasised  the need for staff training.^44^ Incorporating a gender perspective is crucial in preventing bullying and sexual harassment, as it helps to better understand how gender influences the risk of these behaviours and its consequences for both men and women.

Third, Russel and colleagues conclude that reporting systems must be easy to follow and they pinpoint that it is crucial to be able to report harassment behaviours anonymously ^45^ as victims often fear that reporting can have negative consequences for them especially in medical settings.

Fourth, universities must inform their students about how to and the importance of reporting bullying and sexual harassment when it occurs, and secure that no reprisals will be made to those who disclose. In a previous study among university staff, we found that social support from managers buffered negative effects (burn out symptoms) in relation to bullying.^46^ In accordance to those results, universities could develop guidelines in order to support students that have encountered sexual harassment or bullying. Universities could include seminars in the curriculum to increase knowledge for students concerning what is considered acceptable behaviour from staff, patients and relatives and how to report problems. Increased levels of drop out for students and intention to leave health care professions are serious issues, especially in light of the current situation, where the WHO has estimated a shortfall of 10 million health workers by 2030 globally.^47^

## Conclusions

Results show that both male and female students were more often bullied by women. Male students experienced more sexual harassment from women, while female students reported sexual harassment mainly from men. The gender composition in nursing and medical programmes may explain these findings, as mixed-gender environments tend to reduce bullying and harassment. Furthermore, bullying and sexual harassment were linked to increased stress and burnout rates as well as an increased risk of dropping out of education, with the exception of no association between sexual harassment, burnout and intention to quit in male students. The study also demonstrated that nursing and medical students face higher rates of bullying and sexual harassment compared to other academic disciplines. The study highlights the need for further research and interventions to address these problems and support students’ well-being.

### Acknowledgements

The authors of the manuscript would like to thank the participants.

Funding: Forskningsrådet om Hälsa, Arbetsliv och Välfärd, STY-2021/0001

### Conflict of Interest

The authors declare that there is no conflict of interest.
